# Cossham Medical Society 1982-83

**Published:** 1983-07

**Authors:** C. J. Burns-Cox


### Cossham Medical Society 1982–83

Some might say there was too little straight medicine in the programme but we have had rather a good season. Attendances usually exceeded 40 which for so select a society (all meetings were OPEN!) was most gratifying. The presidential address was a crib from Henty on HEROES but the speaker was in deadly earnest – at times racing along, at times rambling discursively. Apparently some Englishmen such as Rajah James Brooke and Thomas Stamford Raffles did succeed in bringing peace and prosperity to their parts of the world although those laden with hindsight try to obscure this issue.

Next Canon Eric Gethyn Jones, the Patrick Moore of Jennerology, from Berkeley, gave us published and new information on the revered Edward Jenner – a delightful talk delivered with that rare mixture of great erudition and super-enthusiasm.

The annual Cocktail Party belied its name by tradition as it is a dinner and dancing evening (the third D omitted in the interests of future party-goers. Perhaps an alcometer should be available).

Professor Stirratt gave his famous talk, published in this *Journal* recently, carefully avoiding any encouragement of home delivery. For those in the know there is a great deal of emotion about babies in Frenchay Health District at the present time.

Tim Lusty, Oxfam Medical Director of Disaster Relief, gave a practical assessment of the place of the doctor in disasters. He plainly had an extraordinary breadth of experience over many years and his gentleness and humility fitted the very best of archetypal English characters — baggy tweeds, ancient brown leather shoes and a full contented face. By the way, would any doctor or paramedical person prepared to join a Disaster Relief Team please contact me at Frenchay?

Recollections of a Magpie by Max Valentine – not in fact a doctor who struts about with bright distinguished plumage but one with many interests amongst which is the collection of artefacts. Earlier years working in Persia started him off with carpets and firearms. As his psychiatric diagnoses became even gentler and less varied over the years so his taste and knowledge of the art world has moved inversely.

The Anti-Slavery Society – does such a creature still exist outside Lyme Regis or the archives? Of course it does and has done so since 1839. Mr. Peter Davies is Secretary-General and he honoured us by telling us of the Society's history and current activities. It is very busy nationally and internationally especially in the U.N. on horrors like debt bondage, serfdom and the plight of the Mauretanians, the difficulties of the Brazilian Indians, child labour in the Phillipines, slavery in Iran and dozens more. You may have seen him this Spring on the television reporting on Mauretania and the Society has also publicised some of the problems of genital multilation. Evil tales indeed from the world stage but the Society makes an earnest attempt to publish and help reduce such cruelties.

Our Last meeting was an all-Bristol event with Mr. and Mrs. Gallop from Bishopston performing 'Victorians through the Magic Lantern': Fantastic lantern slides illuminated by the real thing but no actual limelight for safety reasons.

Throughout the season the secretaries singularly failed to control the president who opened proceedings at all hours (after adjusting the clock to give a semblance of punctuality), sometimes omitted to propose new members but always rememberd the speaker's name. He maintains, and I for one believe him, that this apparent laxity was due to his being frequently overcome by sensibility of the extreme honour of his office. I can reliably inform you that next year's president being Dr. David Skelton, all will run like clockwork and that only the misguided will fail to attend each meeting so exceptional are the speakers-to-be.

